# “Sorry for laughing, but it’s scary”: humor and silence in discussions of Colorectal Cancer with Urban American Indians

**DOI:** 10.1186/s12885-023-11245-y

**Published:** 2023-10-26

**Authors:** Dedra S. Buchwald, Deborah R. Bassett, Emily R. Van Dyke, Raymond M. Harris, Jessica D. Hanson, Shin-Ping Tu

**Affiliations:** 1 Institute for Research and Education to Advance Community Health, Washington State University, 1100 Olive Way, Seattle, WA 98101 USA; 2 Department of Communication, University of West Florida, 11000 University Parkway, Pensacola, FL 32514 USA; 3 Sea Mar Community Health Centers, Seattle, WA 98108 USA; 4 Institute for Research and Education to Advance Community Health, Washington State University, 1100 Olive Way, Seattle, WA 98101 USA; 5 Department of Applied Human Sciences, University of Minnesota Duluth, 1216 Ordean Court, Duluth, MN 55812 USA; 6 Division of General Internal Medicine, Virginia Commonwealth University School of Medicine, Richmond, VG 23298 USA

**Keywords:** Alaska Natives, American Indians, Cancer screening, Colorectal cancer, Ethnography, Focus groups, Gender, Health communication, Health disparities, Qualitative research

## Abstract

**Background:**

Given high rates of cancer mortality in Native communities, we examined how urban American Indian and Alaska Native elders talk about colorectal cancer (CRC) and CRC screening.

**Methods:**

We conducted seven focus groups with a total of 46 participants in two urban clinics in the Pacific Northwest to assess participant awareness, perceptions, and concerns about CRC and CRC screening. Using speech codes theory, we identified norms that govern when and how to talk about CRC in this population.

**Results:**

Our analyses revealed that male participants often avoided screening because they perceived it as emasculating, whereas women often avoided screening because of embarrassment and past trauma resulting from sexual abuse. Both men and women used humor to mitigate the threatening nature of discussions about CRC and CRC screening.

**Conclusions:**

We offer our analytic results to assist others in developing culturally appropriate interventions to promote CRC screening among American Indians and Alaska Natives.

## Introduction

Colorectal cancer (CRC) is a leading health concern for American Indian and Alaska Native communities [1]. Between 2013 and 2017, CRC incidence was 41% higher among American Indians/Alaska Natives than among Whites [2]. In particular, early- and late-onset CRC rates were elevated in this population [3]. Data from the 2001–2018 U.S. Cancer Statistics Database showed that, compared to Whites, early-onset CRC rates were 21% higher in American Indians/Alaskan Natives, and late-onset CRC rates were 15% higher [4]. Other studies have also found increasing CRC incidence in American Indians/Alaska Natives [5, 6], with notable geographic variation. For example, data from the Indian Health Service (IHS) indicated that CRC incidence rates per 100,000 in 2013–2017 ranged from 34.4 in the Eastern region to 96.1 in Alaska [2]. During the same period, incidence declined in some IHS regions (e.g., Great Plains and Pacific Coast), while increasing in others (e.g., Southwest) [7, 8]. Studies in Indigenous populations globally found similarly high rates of CRC when compared to general populations [9, 10].

Among American Indians/Alaska Natives, as in the all-races U.S. population, CRC is the second-leading cause of cancer mortality [11]. But whereas overall cancer mortality in the all-races population declined by 1-3% per year between 1999 and 2008 [12], CRC mortality among American Indians/Alaska Natives increased steadily [5, 13], and it remains higher than among non-Hispanic Whites [14]. Possible explanations for these troubling findings on incidence and mortality include the higher prevalence of obesity and smoking and the lower prevalence of CRC screening among American Indians/Alaska Natives and other Indigenous communities [9, 15]. Notably, this population also has a greater proportion of comorbidities at cancer diagnosis (49.5%), specifically chronic obstructive pulmonary disease and diabetes, relative to non-Hispanic Whites (44.5%, P < 0.05) [16]. An estimated 50-60% of CRC deaths could be prevented if all adults aged 50 and older received routine screening [17]. However, data from the Behavioral Risk Factor Surveillance System (2001–2010) found that American Indians/Alaska Natives had lower CRC screening rates than African Americans and Whites. They were less likely to report screening by colonoscopy or flexible sigmoidoscopy (45%) than both African Americans (56%) and Whites (55%), and they had a lower prevalence of mixed CRC screening than African Americans (61%) and Whites (60%) [15].

These CRC disparities may result from the longstanding effects of colonization, forced acculturation, and scientific misconduct on the Native population [18–24]. Studies with Indigenous populations in Australia found that the significance of a medical mistrust and a lack of awareness of CRC screenings impacted screening behaviors [24–26]. With regard to research misconduct, a 1979 study of alcohol abuse among Inupiat people in Barrow, Alaska, resulted in media publication of results that perpetuated the harmful stereotype of “the drunken Alaska Native” [27]. More recently, the Havasupai Tribe in Arizona won a lawsuit against Arizona State University for using tissue samples for purposes other than the ones originally presented to community members who provided samples [28]. Even though the remaining tissue samples were returned to the community, this case perpetuated a prevalent distrust among American Indians regarding medical research. With regard to continuing CRC disparities, these likely involve the financial, geographic, and cultural barriers to healthcare identified in the literature on American Indians/Alaska Natives [29, 30]. Additional barriers to CRC screening in this population include the competing demands of health, familial, and social issues and a lack of culturally appropriate materials to promote CRC prevention [31].

The goal of the present study was to identify barriers to CRC screening so that our research group could more effectively promote screening uptake in this at-risk population. We conducted focus groups with American Indians/Alaska Natives older than 35 years to discuss their attitudes toward CRC screening. At the time of data collection, guidelines recommended the following modalities for average-risk adults aged 50–75: (a) high-sensitivity guaiac fecal occult blood test (HSgFOBT) or fecal immunochemical test (FIT) every year; (b) sigmoidoscopy every five years plus FOBT or FIT every three years or (c) colonoscopy screening every 10 years [32]. Our study was designed to guide the adaptation of culturally appropriate educational materials for a proposed intervention to increase screening rates. In the present work we report the findings of our focus groups as they relate to culturally appropriate ways of talking about CRC and CRC screening. Given space limitations, we defer for subsequent publication a detailed account of the CRC intervention materials developed as a result of our findings.

## Methods

### Data collection

All study protocols were discussed extensively with site principal investigators and staff at our study sites, and were subsequently approved by the Institutional Review Board of the lead investigator’s institution and our sites. In keeping with the principles of community-based research, we designed and executed the study with input from community partners in Native health, including Native and non-Native clinical staff familiar with the cultural norms of the community in which we worked.

We conducted seven focus groups (two all-male, two all-female, and three mixed-gender) with participants who were patients at two Urban Indian Community Health Clinics in the Pacific Northwest. Research coordinators at both clinics recruited focus group participants by posting flyers at the patient services desk and in the clinic waiting area. Recruitment was also conducted through in-person outreach to participants in clinic programs for elderly and diabetes care. Only adult American Indians/Alaska Natives were eligible for participation.

Our focus group design was based on recommendations in the literature specific to focus groups with American Indians in this region, including a circular arrangement for seating [33]. Researchers and clinic staff collaborated on structuring the discussions. Focus groups lasted 80–120 minutes and were intended to elicit open-ended responses to questions about participants’ understanding of CRC and CRC screening. Questions focused on awareness and knowledge of CRC in Native communities; awareness and knowledge of CRC screening in general; and questions and concerns about CRC screening. Each focus group moderator was a medical doctor with training and experience in leading focus groups with Native elders. A site coordinator or staff member assisted each group by taking notes, coordinating meals, and distributing $25 gift cards as participant incentives. In every focus group, at least one member of the research team present was Native.

Before each group started, the moderators introduced themselves, stating their names, tribal affiliations if Native, and personal backgrounds if non-Native. The moderator then explained the purpose of the overall study and of the upcoming group discussion, and then obtained written informed consent from all participants. Each focus group was audio-recorded. Moderators led discussions of awareness, beliefs, and concerns about CRC and CRC screening. They also presented draft versions of our intervention materials and asked for feedback from participants, who included members of the target demographic for the proposed intervention: urban American Indians/Alaska Natives, aged 50–75 years, at average risk for CRC.

### Data analysis

Audio recordings were transcribed verbatim by a professional transcriptionist experienced in working with Native populations. Any information that might identify individual participants was omitted from transcripts. Our analysis benefited from three decades of interdisciplinary investigations of ways of speaking within various populations, which has produced a body of research known collectively as ethnographies of communication [34]. Among these approaches, we selected speech codes theory, with the goal of describing culturally appropriate ways of speaking about CRC and CRC screening with Native elders.

Speech codes theory is a comprehensive way of understanding a complete communicative interaction. Through careful examination of language in use, this approach enables the identification and explication of a shared way of speaking that has psychological, sociological, and rhetorical significance for the community of speakers. Speech codes theory asserts that in any instance of communication, elements of a speech code are present and identifiable through the communicative conduct (e.g., speech) of its users. This code reveals beliefs, premises, and rules about psychology, sociology, and appropriate communication within a given speech community [35]. Speech codes theory is appropriate for the present study because it permits us to analyze data while remaining open to any construct that appears meaningful to users. Qualitative data analysis is focused on the themes in the participants’ values and beliefs, and speech codes theory allows the qualitative researcher to analyzing a group’s speech code(s) through the lens of its culture [36]. This approach is especially useful when conducting research on previously unexamined or poorly understood concepts of health and wellness among vulnerable populations. It enabled us to identify shared speaking elements that have not previously been noted in American Indians/Alaska Natives.

Our qualitative analyst (DRB), who is trained in ethnography of communication and discourse analysis, read the transcripts and field notes, and used the constant comparative method to distill preliminary themes [37]. She regularly met with both focus group moderators (EVD, SPT) to review early findings and reach consensus on themes. Several of the researchers in this study were American Indians who brought cultural knowledge to bear on the interpretation of focus group data. These meetings included an application of speech codes theory, where discussions centered around exploring local communication practices to better understand meanings and significance of CRC and CRC screening as a way to seek the implications of these remarks [35, 36]. Although it was not feasible, either practically or ethically, to request participant feedback on our analysis of these data, we provided our initial findings to clinical partners at both sites and received validation from clinic staff that our findings were consistent with their interpretation.

## Results

The number of participants per focus group ranged from four to nine, for a total of 46 participants (30 women, 16 men) in seven focus groups. All participants self-identified as American Indian or Alaska Native. Ages ranged from 37 to 71 years, with a median of approximately 60 years. Besides age, race, and sex, no other identifying information (e.g., gender identity, education level, cancer history, or tribal affiliation) were collected.

Overall, we found that male participants were reluctant to talk about CRC. They understood CRC screening as something “embarrassing” and “intimate” that “defies all laws of nature for men,” and they used crude humor to mitigate their discomfort in discussing it. Female participants were more open in discussing these topics, but they also described CRC screening negatively, as “scary,” “embarrassing,” and, for those with a previous history of sexual abuse, as “the end of the world.” We present our results for each gender separately, beginning with the men.

### “I never heard it talked like that before”

One of our primary findings is a clear and consistent response among the male participants that they neither “heard” nor “talked” about this topic in “clinical terms.” The following excerpt presents an exchange that developed when the moderator asked an all-male group what they had heard about “colon cancer”:

Participant (P): I’ve never heard anybody talk about it like that.

Moderator (M): Talk about it like what?

P: Like in clinical terms. “Colon cancer.” They say, oh, your butthole is hurting.

M: You just say your butthole’s hurting?

P: Yeah, or something.. .. I never heard it talked like that before. Don’t think anybody *–* “Oh, I got colon cancer.” Never heard it like that.

Here, the participant stated that “clinical terms” were not used in his peer group; instead, colloquial speech was the standard for discussing medical topics, if they must be discussed at all. To talk about it “like that” was to use an abstract terminology foreign to everyday speech.

The next excerpt presents an exchange that ensued after the moderator asked what the men had heard about CRC screening methods:

P1: You never hear about that stuff.

P2: No.

P1: I mean who wants to talk about it?

M: Nobody wants to talk about it.

P1: Not really.

The phrase “who wants to talk about it?” indicates a norm that discourages discussions of this topic among male American Indian/Alaska Native elders. This norm was echoed by many other participants across focus groups. For example, in another all-male group, the moderator asked if the men ever talked about health issues when they got together:

P1: We talk about hunting and everyday things that people do. But very rare [sic] do they get into health.

P2: Yeah you don’t discuss medical.

M: You don’t ask and people don’t share?

P1: No.

These men described an aversion to speaking about medical topics in general and CRC in particular. Given Native perceptions of hunting as a definitively masculine activity, [11] its mention as a preferred topic of conversation might be a strategic response to an issue that threatened the group’s sense of masculine identity. The perception of CRC screening as threatening to masculinity and heterosexuality was common across both all-male groups.

### “That just defies all laws of nature for men”

Further discussion in the men’s groups revealed beliefs about CRC screening methods that help to explain men’s aversion to talking about CRC and CRC screening. The following excerpt illustrates a conversation that ensued when the moderator asked what the participants knew about CRC screening methods:

P1: They put and stick their finger up your ass.

M: You want to know if they do that?

P1: Yeah.

P2: See that’s what a lot of men think about. That, very truthfully, that’s what a lot of men think about – about that shit.

P1: It’s funny sometimes.

P2: Nobody likes to say it, but that’s what it is.

Here the participants voiced a misunderstanding that is widespread among at-risk populations – namely, that a digital rectal exam is required for CRC screening by FOBT. This misunderstanding presents a major barrier to CRC screening, as participants affirmed when the moderator asked if these beliefs prevented them from getting screened:

P1: For sure, sure.

P2: That just defies all laws of nature for men.

M: It defies all laws of nature for men.

P2: I mean, I don’t know, we’re ignorant that way.

P1: It all depends on how you put it.

P2: It’s a man thing.

P1: Buy you dinner and wine.

P2: Yeah, something like that.

Speech codes theory helps us understand a phrase that could be overlooked as a flippant remark – “that just defies all laws of nature for men” – in two important ways. First, the phrase reveals a cultural belief about what it means to be a man. In this community, being screened for CRC (which the men believed requires a digital rectal exam) would be unnatural for a man. Second, it demonstrates a key coping mechanism: lightening and redirecting the tone and content of the conversation through humor. At this point in the focus group, the men’s responses assumed a joking tone, as suggested by the phrase “buy you dinner and wine.” In other words, CRC screening was equated with a sexual interaction that should be preceded by a romantic dinner and some alcohol.

### “It gets personal if you get down into it”

After the “dinner and wine” remark, one of the participants launched into an anecdote about an embarrassing experience he had while he was hospitalized. His peers spurred him to elaborate:

P1: Hey, I’ll tell you a funny story that happened to me though. Now I had a broken back.

P2: I think it’s time for me to go.

P3: Yeah, it is.

P1: I was stuck in a bed for seven months. Now I had to shit in this fucking bed, they put a plastic mat. .. underneath me, and had a gown that was open in the back.. .. And they had to put one leg up, and wipe me up, wipe me down, put the other leg up, wipe me down, lift, had both my legs up and scoot that mat out and you don’t think that was embarrassing. One of the most embarrassed times of my life. I just laugh and joke around and talking to each other while they’re doing it and I’m going: “oh my God.” It was embarrassing, I mean it was very intimate kind of. Every man choose [sic] that too. That’s all I’ve got to say about colon cancer.

With his anecdote about hospitalization, this participant explained that he used humor, laughing, and joking to cope with his situation. In addition, he drew an explicit connection between “one of the most embarrassed times” of his life and CRC with the statement, “That’s all I’ve got to say about colon cancer.” He suggested that “every man” would use humor to cope with such a humiliating situation. Again, as we note above, remarks that might otherwise be dismissed as a crude attempt to entertain one’s peers help us understand the cultural resources available to Native men when addressing an uncomfortable topic.

In the same focus group, the men expanded on their use of humor to frame a serious situation and put people at ease:

P: Oh I just, I like to joke around.. .. We just, that’s our humor, just to joke around about it, you know.

M: Why joke around about it?

P: Cause it’s the Native humor, just humor, just good medicine.

Here humor, particularly joking, is framed as “good medicine.” In Native cultures, “good medicine” is a positive, culturally-based construct that might include any number of elements that help a person achieve or maintain wellness.

However, another type of humor also appeared in discussions of CRC: teasing, which participants described but did not name. Unlike joking, teasing was not described as “good medicine,” but as emasculating and humiliating. For example, in the next excerpt, participants in the same focus group revealed that if one of them were diagnosed with CRC, or even received CRC screening, he would never mention it to his peers. They suggested that doing so would invite teasing from other men and would be threatening to their sexuality:

M: So do you think. .. people could be having colon cancer and they’re not talking about it for those reasons?

P1: Yes.

P2: Yeah, cause who’s going to talk and say.

P3: Wait, what was that, you’re talking about your butthole?

P1: Oh, you got cancer in your butt, right.

P2: Yeah, who’s going to say that?

P1: I don’t know.

P3: Yeah, who’s going to say that?

P2: Maybe [Name] or the bisexuals.

P1: Hey, I got cancer.

P2: They probably get a good joke out of it.

P1: Yeah.

P2: But those that are straight they won’t talk about it, you know what I mean.

It is clear that for these men, “those that are straight. . won’t talk about” CRC or CRC screening because of shared norms that prohibit discussing certain parts of the anatomy. To violate this norm would risk teasing from other men about being bisexual – in other words, not straight. Indeed, the persistence of homophobia has been well documented in Native communities [12].

The perception of CRC screening as threatening to a man’s sense of masculinity was often reiterated. For example, in another focus group, a male participant provided a possible reason why colon cancer occurs at higher rates among Native men:

P: In my tribe, a lot of the men don’t want that invasive procedure of a colonoscopy or even the procedure to test the prostate because it’s invasive and they feel that it’s emasculating and I think that that’s maybe a reason that Native men have perhaps a higher rate of colon cancer.

This participant reaffirmed the belief that an “invasive procedure” – colonoscopy, one of the recommended methods for CRC screening – would threaten a Native man’s sense of masculinity.

Another all-male discussion provided especially rich data on the use of humor to respond to a “touchy” and “personal” topic:

M: Anything else to add on the subject of colon cancer and colon cancer screening?

P1: I think we about said it all.

M: You think you said it.

P2: I think it’s a touchy subject.

P3: Yeah.

P4: It gets personal if you get down into it.

P3: Yeah, it does.

P1: And that’s just four of us that are saying this. Imagine if there’s 20, this place would be laughing, and then it would get quiet like it just did.

According to this participant, when the men’s discussion was dominated by joking and laughter, followed by silence, their behavior identified a topic as difficult for the group and outside the norms of their speech code. Additionally, the statement “I think we about said it all” signals their aversion to continuing the CRC conversation. Although these men used jokes and blunt language to deflect and render slightly more palatable such an uncomfortable topic, they would much rather avoid it completely. The phrase “it gets personal if you get down into it” suggests that delving into the topic was uncomfortably intimate for discussion in this group.

### “Sorry for laughing, but it’s scary”

Although our findings on norms that govern discussions of CRC relate primarily to Native men, we identified analogous strategies among Native women. In one all-female focus group, humor again emerged as a way to mitigate the discomfort of talking about the “scary” topic of submitting to a rectal exam. One woman stated that both of her parents died of CRC, and that she worried she might also develop the disease. Nevertheless, she revealed, she was extremely reluctant to be screened for CRC. Her explanation surfaced in the following exchange:

P: What’s the word? Genetic. So I’m really scared, but I don’t want nobody going up my butt. That’s scary. [Laughs.] I mean nothing touches my butt. You know what I’m saying? And I’m like: how can I let somebody do that? You know, if I can’t, it’s just like, oh my goodness, that’s scary.

M: All right. Thank you for sharing.

P: Sorry for laughing, but it’s scary.

Later in the same focus group, the moderator followed up the topic of reluctance to screen:

M: Do you think that’s a common experience: delaying the prevention or the screenings? Or is that just an experience for somebody who’s –

P: I think it’s a common experience because when you’re Native you don’t trust.. .. For our history, as Natives, we’ve always had bad experiences with the government and the Western medicine.. .. So getting us in this circle is a miracle – getting us to discuss this stuff. For me to be here, it took many moons to get me here, to want to be willing to participate right now.

In this exchange, a Native woman concisely explained that the history of abuse and mistreatment of Native people by the U.S. government and the U.S. medical establishment has made many American Indians/Alaska Natives unwilling to participate in medical research, or even to discuss medical topics. Another female participant in the same group echoed remarks made in an all-male group when she characterized CRC screening as “embarrassing”:

P: I think that with this particular subject, that it’s – we’re kind of like an embarrassed people. I know this is embarrassing to do this and so it’s something that you’re going to put off, especially having some – I think it would just – it’s just embarrassing. I’ve never had to do that. I mean I’ve had a colonoscopy, but I haven’t had to do the poop on a stick or whatever.

M: So is it all embarrassing or is it the poop on a stick that’s embarrassing?

P: I think, just, it’s all embarrassing.

In the same group, participants went on to discuss the invasiveness of CRC screening procedures and their lack of choice regarding testing modalities. The available procedure was described as a “foreign body” and “scary.” Participants agreed that a history of trauma in Native communities, especially sexual trauma, meant that screening procedures could be further traumatizing and might trigger negative emotions. One participant said that doctors did not realize how much they were asking for when they advised survivors of sexual trauma to obtain screening for CRC, cervical cancer, or breast cancer. Many participants said that their personal history of sexual trauma, combined with their providers’ insensitivity to that history, made medical encounters difficult for them.

In one of the mixed-gender focus groups, a female participant expressed intense discomfort with being bodily exposed in front of a medical doctor:

P: Yes, showing your parts, I mean, really, that’s the end of the world. You’re completely disrobed and you’re vulnerable.

This woman equated being “disrobed” for examination with “the end of the world” and being “vulnerable.” Her response is consistent with descriptions offered in all-female groups. It illustrates how difficult it is for many Native women to undergo a procedure that is typically considered unremarkable by clinicians.

Although female participants were markedly more willing than male participants to talk about CRC and CRC screening, their responses revealed a different reason for avoiding screening. Whereas the men indicated that their aversion was motivated by a perceived sexual connotation and subsequent threat to heterosexual masculine identity, the women’s responses suggested that their reluctance was rooted in a fear of feeling vulnerable or triggering traumatic memories of sexual abuse. Nevertheless, both genders used humor to talk about CRC screening: the men used joking and the women used laughter when faced with this potentially distressing topic.

## Discussion

It is hardly remarkable that contemporary American Indians/Alaska Natives and other Indigenous communities mistrust the Western medical establishment, given the history of unethical, abusive, and neglectful practices inflicted on their communities by the federal government in general, and by medical researchers in particular [18–24, 27, 28]. This history of mistrust has a negative impact on the implementation of effective health interventions, as has been seen in previous research on cancer treatment in Indigenous communities globally, which found that this population expressed significantly higher levels of mistrust in the healthcare setting and lower levels of satisfaction with healthcare relative to Whites [18, 25]. In addition to these previous studies, our research adds to the understanding of CRC screening barriers in American Indian/Alaska Native communities by providing an understanding of the role of gender norms, threats to masculinity, loss of privacy, embarrassment, and histories of sexual abuse.

Although the findings reported here represent a small group of Native adults in the U.S., they are consistent with the results of similar studies on barriers to CRC screening among ethnic minorities in both the U.S. and the U.K. For example, a U.K. study in 2008 found that ethnic minorities overall were less likely than Whites to get screened because of “shame” and “embarrassment”[38]. A U.S. study in 2011 found that African American men were reluctant to get screened because of “the perceived sexual connotation” of the procedure [39], a finding markedly similar to our own regarding Native men. Similarly, a 2013 study with American Indians under age 50 found that Native men did not like to talk about CRC, partly because of a “homophobe type thing” and partly because they might be viewed as “a softy” [40]. Finally, a study that focused on the role of “masculinity barriers” to medical care in racially diverse populations found that American Indian/Alaska Native men were least likely to report CRC screening intent (51.1%) compared with African American (68%) and White men (64%) (*p* < 0.001); moreover, Native men who exhibited more masculinity-related barriers to care were less likely to have CRC screening intent compared to other racial groups [41].

Findings from this analysis were based on consistent communicative patterns observed across all seven focus groups. After considering our findings in the aggregate, we were able to distinguish elements of the speech code used and understood by our focus group participants. These findings are consistent with the limited extant research on barriers to CRC screening in Native populations. For example, in a similar study involving interviews with community leaders and providers, barriers included historic distrust, repulsion by screening methods, and gender roles; participants also identified a need for culturally appropriate educational materials [42]. A review of cancer screening interventions in First Nations communities in Canada found similar results, where the use of multiple culturally appropriate strategies were the most effective in increasing cancer screenings [10]. Other studies have found that community members had little knowledge of CRC and did not discuss it openly [25, 40], therefore providing culturally appropriate information about screenings may be impactful. To our knowledge, the present study is the first to identify a shared way of speaking about CRC and CRC screening among Native elders.

Our analyses of data from seven focus groups indicate the existence of a speech code for discussing (or not discussing) CRC in this population. We offer the following norms to elucidate how urban American Indians/Alaska Natives talk about this and other sensitive medical topics: (1) a man does not talk about emasculating topics such as CRC or CRC screening; (2) if a man must talk about emasculating topics, he will use colloquial language and humor; and (3) joking and laughter followed by silence might indicate discomfort with the topic or premise of the discussion. Among female participants, the fact that a group of Native women could discuss CRC was described as “a miracle.” Whereas previous research has identified “embarrassment” as a barrier to CRC screening for Native women [40], our all-women focus groups revealed a powerful and traumatic reason for this embarrassment: a history of sexual abuse.

To address these realities, speech codes theory helps us answer a critical question: What is a suitable way for researchers and medical practitioners to engage in dialogue on CRC with Native elders? This challenge offers a pathway for effective communication about CRC prevention: using elements of a given speech code to introduce changes in that code. The unique contribution of speech codes theory to health interventions lies in its understanding of speech codes as human and social constructs that are open to change. If our analysis simply concluded that CRC and CRC screening were inappropriate to discuss with urban Native elders, the public health outlook would be dismal indeed. However, understanding a speech code as a dynamic construct permits us to introduce new concepts to Native communities in culturally appropriate ways. By working with the community to expand locally-held notions of what is considered appropriate to talk about, and with whom, we explicitly address a health crisis in Native populations.

The critical role of humor in our focus groups reflects previous commentary on Native culture. Vine Deloria, an American Indian author, theologian, historian, and activist, wrote, “One of the best ways to understand a people is to know what makes them laugh. . In humor life is redefined and accepted. Irony and satire provide much keener insights into a group’s collective psyche and values than do years of research” [43]. Indeed, a rich body of literature attests the special place of humor in American Indian life. As American Indian poet Paula Gunn Allen wrote, “Humor is widely used by Indians to deal with life. Indian gatherings are marked by laughter and jokes, many directed at the horrors of history, at the continuing impact of colonization, and at the biting knowledge that living as an exile in one’s own land necessitates” [44]. Humor is widely known to be impactful in health care, both as a way for a health care provider to interact with patients [45–47] and as a way for patients to cope with stressful health situations and interactions (e.g., “coping humor”), particularly in regards to cancer screening and diagnoses [48–50]. Humor may also play a role in healing by promoting positive emotions and beneficial physiological changes [48, 51***Recommendations and future work***.

We recommend that future efforts be devoted to community education on CRC screening recommendations and specific deterrents to screening so that misconceptions can be promptly addressed during conversations about CRC – both in clinical settings and in public health promotion. In developing such interventions and programs, we also recommend the inclusion of a question-and-answer component in focus groups if the topic warrants it. Our experience suggests that, in focus groups that include American Indian/Alaska Native elders and address sensitive topics such as CRC, participants and moderators should be congruent in culture, gender, and age whenever possible. In addition, it is important to provide home testing kits to increase CRC screening in a private, convenient way. For example, one study with American Indian/Alaska Native community members included direct mailing of an FIT kit. Among participants who were mailed FIT kits without outreach, 16.9% returned the kits – a significant increase over usual care (P < 0.01). Notably, among participants who returned FIT kits, 23.6% had a positive result and were referred for colonoscopy, and 59.0% of this subgroup completed colonoscopy [52].

Given the importance of humor in mitigating the discomfort that patients feel when they discuss CRC screening, clinicians and public health workers who promote screening need to be receptive to the use of humor in patient interactions. They should be willing to engage with patients through humor while remaining aware that humor coupled with silence might signal discomfort. In particular, clinicians who encounter laughter and joking followed by periods of silence and deflection of questions should proceed with sensitivity and caution. Clinicians should also acknowledge the powerful cultural role of humor as medicine among Native patients. Previous research has also found that programs promoting CRC prevention in American Indian/Alaska Native communities should focus on reasons for getting screened and the role of culture in preventing CRC [31].

We acknowledge the limitations of the present work. Although the focus group moderators for the all-female groups were gender-concordant, moderators for the all-male groups were not. Nevertheless, we appeared to create a supportive research environment in which the men felt comfortable discussing sensitive topics. We also recognize that the perceptions of our sample of 46 focus group participants cannot be generalized to all urban Native elders. However, we feel that the quality of the data collected offsets this limitation. We recommend additional, cumulative research to develop more robust analyses of speech codes that will contribute to more effective cancer screening interventions for urban Native elders.

## Data Availability

The datasets generated and analyzed during the current study are not publicly available due to tribal sovereignty regulations. Data can be made available by contacting the corresponding author and by formally applying to the relevant tribal councils and tribal review boards for access.
